# An Automated Image-Based Multivariant Concrete Defect Recognition Using a Convolutional Neural Network with an Integrated Pooling Module

**DOI:** 10.3390/s22093118

**Published:** 2022-04-19

**Authors:** Bubryur Kim, Se-Woon Choi, Gang Hu, Dong-Eun Lee, Ronnie O. Serfa Juan

**Affiliations:** 1 Department of Robot and Smart System Engineering, Kyungpook National University, 80 Daehak-ro, Buk-gu, Daegu 41566, Korea; brkim@knu.ac.kr; 2 Department of Architectural Engineering, Daegu Catholic University, Hayang-ro 13-13, Hayang-eup, Gyeongasan-si 38430, Korea; watercloud@cu.ac.kr; 3 School of Civil and Environmental Engineering, Harbin Institute of Technology, Shenzhen 518055, China; hugang@hit.edu.cn; 4 School of Architecture, Civil, Environment and Energy Engineering, Kyungpook National University, 80 Daehak-ro, Buk-gu, Daegu 41566, Korea

**Keywords:** concrete cracks, convolutional neural network, delamination, multivariant defects, spalling, surface crack

## Abstract

Buildings and infrastructure in congested metropolitan areas are continuously deteriorating. Various structural flaws such as surface cracks, spalling, delamination, and other defects are found, and keep on progressing. Traditionally, the assessment and inspection is conducted by humans; however, due to human physiology, the assessment limits the accuracy of image evaluation, making it more subjective rather than objective. Thus, in this study, a multivariant defect recognition technique was developed to efficiently assess the various structural health issues of concrete. The image dataset used was comprised of 3650 different types of concrete defects, including surface cracks, delamination, spalling, and non-crack concretes. The proposed scheme of this paper is the development of an automated image-based concrete condition recognition technique to categorize, not only non-defective concrete into defective concrete, but also multivariant defects such as surface cracks, delamination, and spalling. The developed convolution-based model multivariant defect recognition neural network can recognize different types of defects on concretes. The trained model observed a 98.8% defect detection accuracy. In addition, the proposed system can promote the development of various defect detection and recognition methods, which can accelerate the evaluation of the conditions of existing structures.

## 1. Introduction

Nowadays, assessing structural health conditions is necessary because of the numerous issues and failures of some structures. Reference [1] developed a method for detecting and localizing single and multiple damages on bridges by analyzing the vibration characteristics using a mode shape component-specific damage index. Another previous study is reported by [2] and describes a strategy for detecting, identifying, and quantifying damage in order to categorize diverse categories into broad non-parametric and parametric classifications. The authors of [3] described a method that utilized MEMS-based sensors in conjunction with an enhanced autoregressive model for structural monitoring that was especially applicable to towers. Among the parameters that contribute to the deterioration of the components of a structure are various defects on concrete, such as surface cracks, delamination, and spalling [4]. The authors provide background information on delamination in concrete, which is frequently produced by a high air content that becomes trapped behind a tight power-troweled finish on the surface, while spalling is caused by a variety of factors, including poor concrete quality, insufficient curing, and poor finishing processes, to mention a few [4,5]. The individual discussion of these parameters is presented in the related works section. In the past, the evaluation and investigation of the health condition of structures were conducted manually with human intervention. However, when humans provide the assessment, subjective instinct is used to perform tasks such as crack analysis, and the results are frequently time-consuming and more prone to error.

Despite the existing protocols on detailed visual examinations for manual assessments of concrete, humans still use psychophysical measurements in evaluating image quality, which is based on the human perception of visual information [6]. Given these human constraints inherent in manual inspections, the results may be inefficient and cause serious problems that contribute to the continuous deterioration of structures.

The implementation of computer vision can overcome the said drawbacks and can automatically recognize and classify different types of defects on concrete. Computer-based assessments provide superior advantages, especially in terms of recognition and classification. The following studies employed computer-vision-based systems for classification applications. In addition, these research works utilized convolutional neural network (CNN) models to immediately preserve and secure the structural stability of buildings or structures. Reference [7] used a surface crack detection approach, which included convolution and pooling layers for a concrete image dataset applied to image processing and deep learning techniques. Another study [8] implemented a concrete crack detection and monitoring scheme founded on a deep-learning-based multiresolution analysis to impose an automatic crack type recognition based on CNN. Moreover, other research [9] implemented a CNN-based automated pavement crack identification model to distinguish between defective and non-crack concrete.

The main objective of this study is to improve the existing research. The proposed scheme in this paper is the development of an automated image-based concrete condition recognition technique that can be used, not only to categorize non-defective concrete into defective concrete, but also to recognize multivariant defects such as surface cracks, delamination, and spalling. The proposed multivariant defect detection neural network architecture is based on a convolutional model capable of recognizing various types of defects in concrete. In addition, the suggested system aims to aid the development of different fault detection and identification techniques and expedite the assessment of the state of existing structural components.

The remainder of this study is structured as follows. Section 2 discusses the literature related to the proposed method. Section 3 details the methodology of the proposed algorithm. Section 4 includes experiments and discussions of the results. Finally, Section 5 concludes this study.

## 2. Review of Related Works

### 2.1. Defect Detection

The following are certain existing studies related to the proposed study. Reference [10] used deep CNNs to annotate a concrete dataset for noncontact concrete detection. However, viewing the resulting image using the said neural network is difficult when it is compared with the original raw image. Rather than relying on conventional methods for assessing cracks in concrete structures, digital image processing and 3D scene reconstruction were used to achieve the dataset image for resizing and reconstruction in another study [11]. However, the study did not provide a comparative analysis of both the original and resulting images. Other research implemented a 2D mesoscale model for a concrete base, which used an interface element with a high aspect ratio [12]. Such a study was purely a simulation process conducted in Monte Carlo; thus, the said model might produce different output consistencies. Moreover, reference [13] provided a multiresolution analysis for a wavelet-based method coupled with deep learning to efficiently monitor cracks in concrete. However, the dataset provided and used to classify the crack to non-crack concrete was limited. Likewise, in reference [14], the said concrete detection or classification was only limited to two conditions (non-crack and crack concrete). An experimental research work presented in [15] utilized CNN to identify tiny surface cracks on ceramic tiles. However, although it provided an effective scheme for classifying the defects, the model presented is limited to only two conditions. Thus, this study aims to enhance such existing studies.

### 2.2. Types of Concrete Defects

Cracks in structures primarily result from poor design and construction. Additionally, these faults have a detrimental effect on the structures’ health condition and are even hazardous to humans in cases of accidents. In this study, only three types of concrete defects were selected, as presented in the following.

#### 2.2.1. Surface Crack (SC)

**Figure 1:**
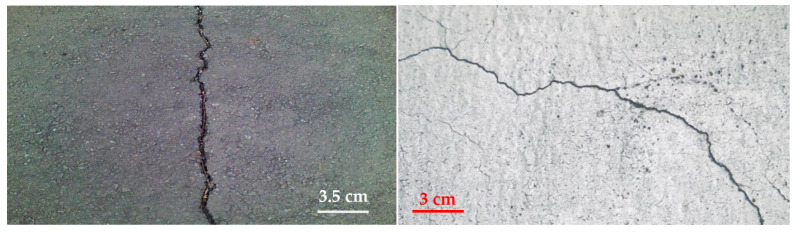
Sample images of concrete with surface crack defects.

As shown in Figure 1, surface or small opening cracks are common defects in concrete structures with a scale bar to provide the dimension of the surface. Typically, these are caused by a combination of premature drying, overloading, shrinkage during drying, temperature variations, chemical reaction exposure, weathering, differential settlement, and other degradation processes [16]. Generally, cracks make concrete and structures more vulnerable to damage from outside forces, speed up the aging process, and weaken the structure’s mechanical strength [17]. Additionally, cracks limit a structure’s capacity to absorb stress, which might result in a structural collapse. If cracks form, their effect on the strength of the structure should be evaluated and monitored to ensure the concrete’s health.

#### 2.2.2. Delamination (DM)

**Figure 2:**
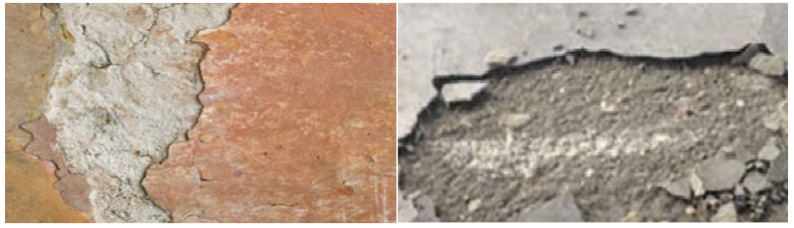
Sample images of concrete with delamination defects.

**Figure 3:**
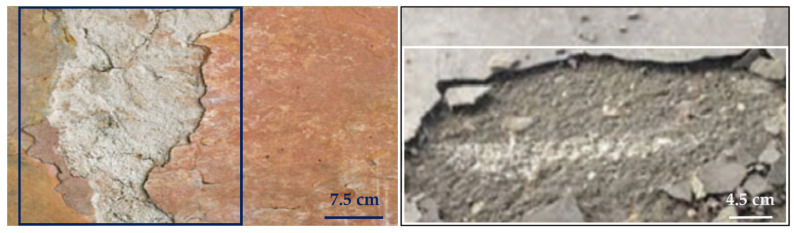
Sample images of delamination defects in concrete with scale bar.

Concrete delamination occurs when the cement paste layer separates from the slab body, resulting in an unbonded concrete layer [18]. This problem happens most commonly with troweled concrete during the early spring and late fall, whenever concrete is laid on a cool substrate. However, depending on the concrete and the finishing techniques utilized, such a separation might occur at any time. Once delamination is not prevented, it begins to spread on the whole structure, and the concrete surface performance will be badly affected [19]. Sample images of the delamination of concrete are shown in Figure 2, while Figure 3 provides a scale to identify the dimension of the surface.

#### 2.2.3. Spalling (SP)

**Figure 4:**
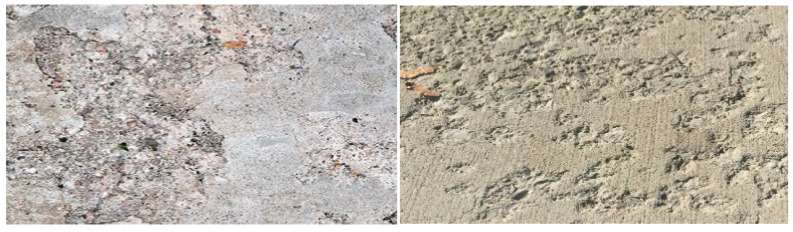
Sample images of concrete with spalling defects.

**Figure 5:**
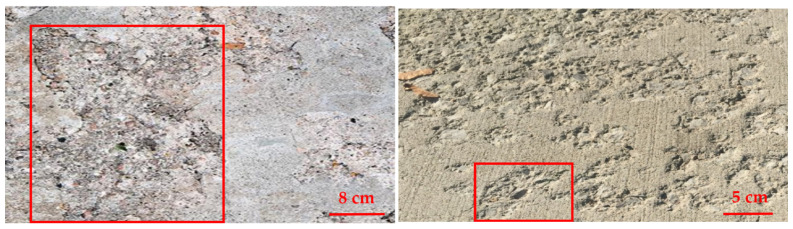
Sample images of spalling defects in concrete with scale bar.

Spalling is the cracking and delamination of concrete from the substrate [20]. Spalling can occur due to freeze-thaw cycles, alkali silica reactions [21], or exposure to fire. Spalling may be dangerous because it results in falling debris. It may also speed up and spread through the structure, making it more unstable. In addition, during temperature exposure, spalling occurs when layers or chunks of concrete break away from the surface [22]. The effect of spalling will become more vulnerable to corrosion particularly when the reinforcement in the concrete is exposed; corrosion will eventually lead up to the failure of steel and may even cause the collapse of the entire structure [23]. Figure 4 shows examples of how spalling looks like on concrete, and Figure 5 provides the same image with a scale bar to see the dimension of the surface.

## 3. Proposed Method

**Figure 6:**
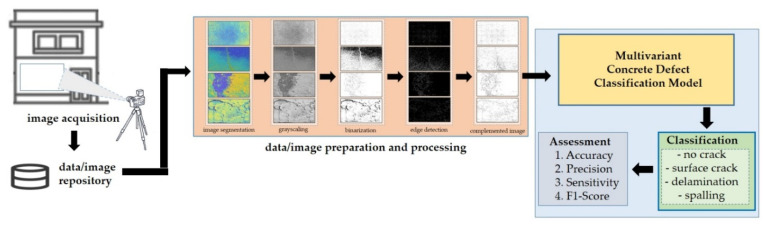
The proposed scheme of the study.

Figure 6 shows the proposed scheme. The following sections include descriptions of each step of the proposed work. The input images were subjected to preprocessing to improve their quality. Subsequently, the images were enhanced using various image processing techniques. Moreover, a CNN approach for automated image classification was used to evaluate the classification accuracy of the testing images.

### 3.1. Dataset

**Figure 7:**
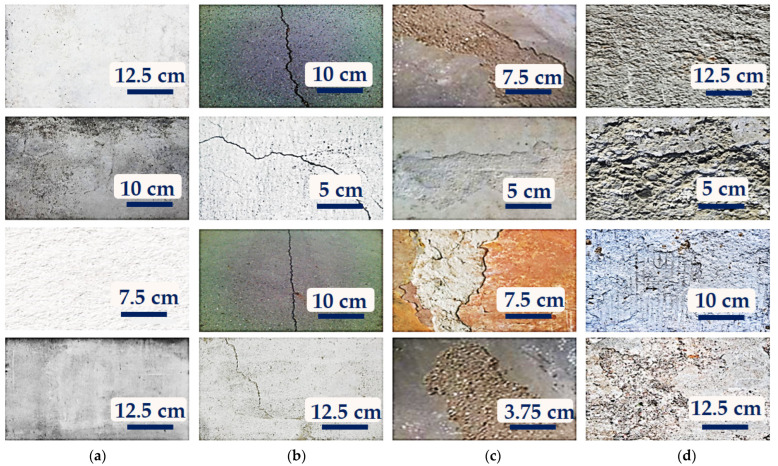
Samples of the dataset images used for this study: (**a**) non-crack; (**b**) surface crack; (**c**) delamination; and (**d**) spalling.

The proposed model is trained using a set of non-crack (NC) and cracked concrete images. The defective concrete images are composed of three variants: surface crack (SC), delamination (DM), and spalling (SG). The dataset is composed of 3650 images collected from various structural establishments in Daegu City, Republic of Korea. The dataset is divided into a training set (70%) and a testing set (30%). Table 1 shows the dataset’s breakdown. Figure 7 shows samples of the images used for this study.

### 3.2. Image Processing

**Figure 8:**
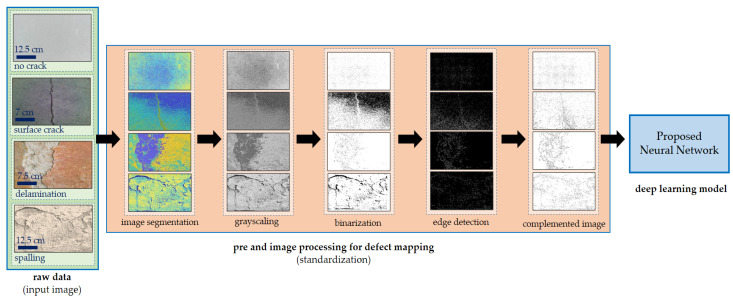
Proposed algorithm.

Figure 8 shows the image enhancement process used in this study. The details of each digital image processing technique used in the proposed algorithm are listed below.

Step 1. Initially, image segmentation is employed to convert the input image into something more manageable to analyze [24]. This study uses image segmentation for concrete images. Features or attributes were extracted with a *k* value of 3.

Step 2. The grayscale level of an image is used to eliminate the hue and saturation content from the image but keep the luminance [25,26]; grayscale images employ a single value per pixel known as intensity or brightness [27]. In this study, changing an image to grayscale better changes its aspect, because it changes the depth of contrast at a pixel value, resulting in a more noticeable appearance.

Step 3. The image binarization process replaces all values greater than a globally determined threshold while converting the image to a binary image with 1 s and all other values with 0 s [28]. The default Otsu approach is employed to minimize the variation of the thresholded black and white pixels.

Step 4. The edge approach identifies the most essential edge aspects of an image and serves as a filter to improve the image [6].

Step 5. Color complement. Each color channel in the produced image is complemented by the corresponding color channel in the original image [29]. The dark areas become lighter, or the color is reversed.

### 3.3. Image Classification

The factors considered for the image classification are listed in Table 2.

With the advent of powerful electronics devices, the training time and hardware requirements are no longer a hindrance to the advancement of the neural network [30]. Furthermore, data scarcity may be addressed by data augmentation [31]. Finally, as observed in [32,33], the majority of systems are provided with configuration capabilities.

#### 3.3.1. CNN Architecture

CNNs are deep neural networks frequently used in image classification [34]. A similar method is implemented by [35], but the application is for the steel frame damage with the inclusion of a computer vision method. The study [36] presents how to utilize CNN and transfer learning to automatically classify and separate cracks on masonry surfaces. They consist of convolutional layers equipped with an activation function, a pooling function for assessing input characteristics, and connected layers for classification [37]. The pooling layers enable the downsampling of feature maps by enumerating the features present in patches of the feature map [38].

As the core components of the neural network that performs the convolutional operation, the set kernel filters provide the link between the input features [16]. The expression for the mathematical relationship of the convolutional layer for each location *U_y_* of the output *y* is shown in Equation (1):

*y (U_y_)* = Σ*w* (*U_P_*)∙*x*(*U_y_ + U_P_*) (1)

where *x* is the input variable, *w* denotes the filter, *P* denotes the field in the convolutional layer, and *U_P_* denotes the location inside the field *P*. The inputs to a 2D CNN layer may be observed as a collection of 2D matrices with discrete channels based on their picture representations. The convolutional layer incorporates many filters capable of scanning inputs and creating output mappings. Multiple filters in the convolutional layer are capable of scanning inputs and providing output mappings. When *M* inputs and *N* outputs are present, *M N* filters are required to accomplish the convolutional operations. In this study, the sole purpose of the neural network is to verify the accuracy of the classification of the processed images with the seam-carved output images and consider the abovementioned factors.

#### 3.3.2. VGG16

VGG16 is the most often used CNN variant. It comprises a total of 16 layers, 13 of which are convolutional and three are completely linked [39]. It uses ReLU as an activation function to improve its nonlinearity, whereas the softmax function is used for classification in the final layers. The implementation is described below. The model is initialized by the following specific sequence:

1. 2 × convolution layers with 64 channels in a 3 × 3 kernel with the same padding;
2. 1 × maxpool layer with a 2 × 2 pool size and a stride of 2 × 2;
3. 2 × convolution layers with 128 channels in a 3 × 3 kernel with the same padding;
4. 1 × maxpool layer with a 2 × 2 pool size and a stride of 2 × 2;
5. 3 × convolution layers with 256 channels in a 3 × 3 kernel with the same padding;
6. 1 × maxpool layer with a 2 × 2 pool size and a stride of 2 × 2;
7. 3 × convolution layers with 512 channels in a 3 × 3 kernel with the same padding;
8. 1 × maxpool layer with a 2 × 2 pool size and a stride of 2 × 2;
9. 3 × convolution layers with 512 channels in a 3 × 3 kernel with the same padding;
10. 1 × maxpool layer with a 2 × 2 pool size and a stride of 2 × 2.

ReLU activation is added to each layer to avoid the passing of negative values to the next layer. Then, upon creating all convolutions, the data are passed to the dense layer:

11. 1 × dense layer with 4096 units;
12. 1 × dense layer with 4096 units;
13. 1 × dense softmax layer with 2 units.

#### 3.3.3. Architecture of the Proposed Model

**Figure 9:**
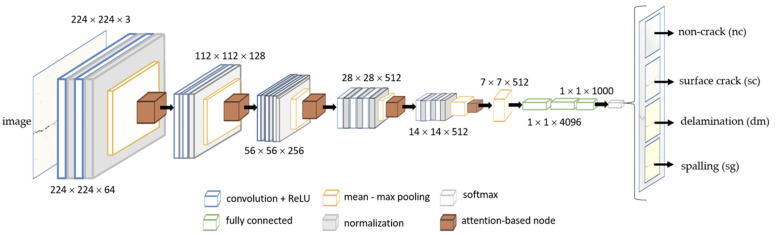
The proposed model structure using a multivariant defect recognition neural network.

As part of the objectives of this study, a CNN is utilized for the classification of defective and non-crack concrete images. The multivariant defects on concrete are categorized into three variants: surface crack, delamination, and spalling. The architecture of the convolution-based multivariant defect classification neural network is presented in Figure 9. The network is a reconfigured VGG16 with an integrated max–mean pooling layer and attention-based [39,40] network node, which aims to further extract the significant feature maps of the image dataset.

##### Integrated Max–Mean Pooling Layer

**Figure 10:**
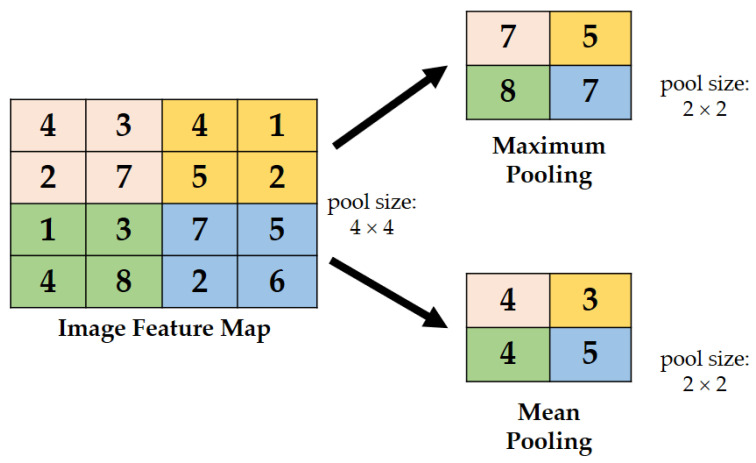
Illustration of max–min pooling.

Generally, the disadvantages of a maximum and mean pooling are that they may lose information present in the image. However, an integrated or combined function may avoid such loss of significant information. Figure 10 shows the representation of the max–mean pooling concept used in this study. The pooling layer is an integrated maximum and mean pooling concept used to evaluate all components in the pooling areas to reduce variance while retaining background information [41] and only captures the greatest activation as a region’s representative feature [42]. For this study, the implementation works as follows. For example, when a 2 × 2 convolutional layer is extracted to the pooling layer, the maximum and average pooling layers are utilized and combined into an integrated max–mean pooling layer module before being extracted to the 1 × 1 convolutional layer.

##### Attention-Based Network

The main purpose of an attention-based network is to recognize multiple objects in images [42]. The method aims to simulate cognitive attention. The effect boosts/enhances some features of the input data while reducing others—the idea being that the network should provide a greater emphasis on that small but critical segment of the data [43].

**Figure 11:**
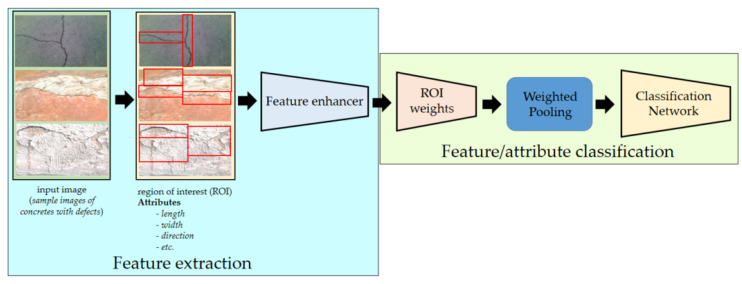
Attention-based network that recognizes multiple objects in an image.

In this study, the attention-based node employs the max–mean pooling technique to realize the means of the link of the network, which determines the mean based on the channel axis to achieve the maximum possible performance. Figure 11 shows the architecture inside the attention-based network used in this study. Under the feature extractor block, the region of interest scheme maps the features of each image. Before feeding information to the fully connected layers, the feature or attribute classification computes the weight and aggregate of the ROI features and performs multilabel feature/attribute classification.

### 3.4. Implementation

The experiments in this study were conducted using the MATLAB platform with a reconfigured CNN-based model. As baselines for categorizing concrete damage recognition, CNN models (i.e., ResNet50, VGG16, and MobileNetV2) were used. The experiments were conducted using a workstation equipped with a GPU (NVidia GTX1080-Ti 11G) and CPU (Intel Core i7-1065G7 CPU, 2.60 GHz × 18). Preliminary testing was conducted using VGG16, ResNet50, and MobileNetV2 to determine the ideal architectures for the concrete damage dataset. The dataset was divided into training and test data in a 70:30 ratio for the experiments performed in this study. The training and testing datasets were thus divided into 2555 and 1095 images, respectively. The validation loss per epoch was monitored and weight variables were adjusted when the validation loss decreased throughout the training phase to ensure that the experimental models performed optimally. Thereafter, the testing dataset was subjected to performance evaluations and assessments.

Below is the layer implementation of the proposed model for the training of 5000 iterations.

layers = […
   imageInputLayer ([227, 227, 3])
   convolution2dLayer(5, 20)
   reluLayer
   maxPooling2dLayer (2, ’Stride’, 2)
   fullyConnectedLayer (2)
   softmaxLayer
classificationLayer];
   options = trainingOptions(‘sgdm’, …
   ‘ExecutionEnvironment’, ‘cpu’, …
   ‘MaxEpochs’, 100, …
   ‘ValidationData’, {XValidation,YValidation}, …
   ‘ValidationFrequency’,1000, …
   ‘InitialLearnRate’, 1 × 10^−4^, …
   ‘GradientThreshold’, 1, …
   ‘Verbose’, false, …
   ‘Plots’, ‘training progress’);

Table 3 shows the hardware specifications of the deep learning computer we used for the simulation using the MATLAB platform.

## 4. Discussion of Results

**Figure 12:**
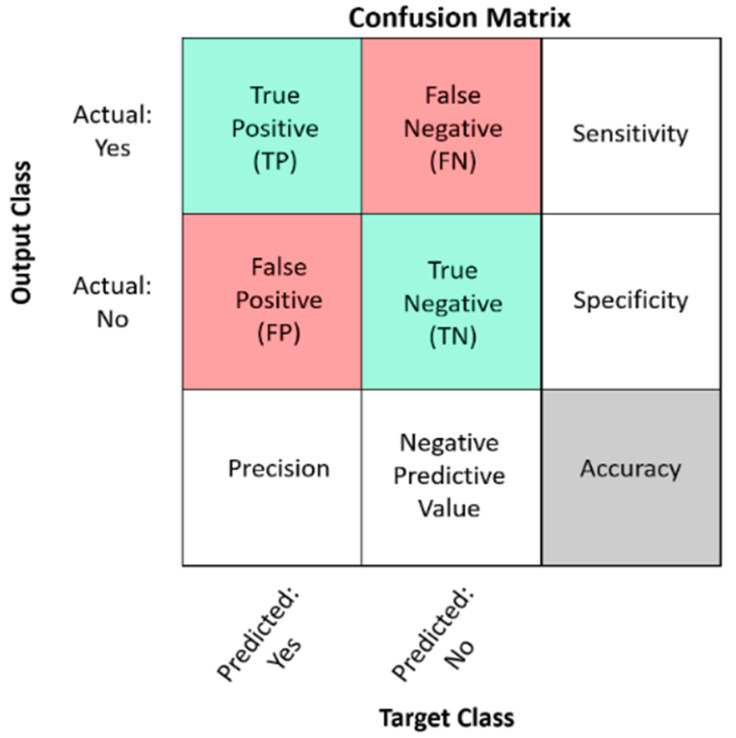
Legend of the confusion matrix.

Different parameters were employed, including accuracy, precision, sensitivity, and F1-Score, to demonstrate the significance and performance of this paper’s proposed model. The following shows the description of each parameter as shown in Figure 12.

The following equations of the parameters used in this study are based on the confusion matrix interpretation.

(2) $$\mathrm{Accuracy}=\frac{TP+TN}{TP+TN+FP+FN}$$

(3) $$\mathrm{Precision}=\frac{TP}{TP+FP}$$

(4) $$\mathrm{Sensitivity}=\frac{TP}{TP+FN}$$

(5) $$F1-\mathrm{Score}=\frac{Precision*\;Sensitivity}{Precision+Sensitivity}$$

**Figure 13:**
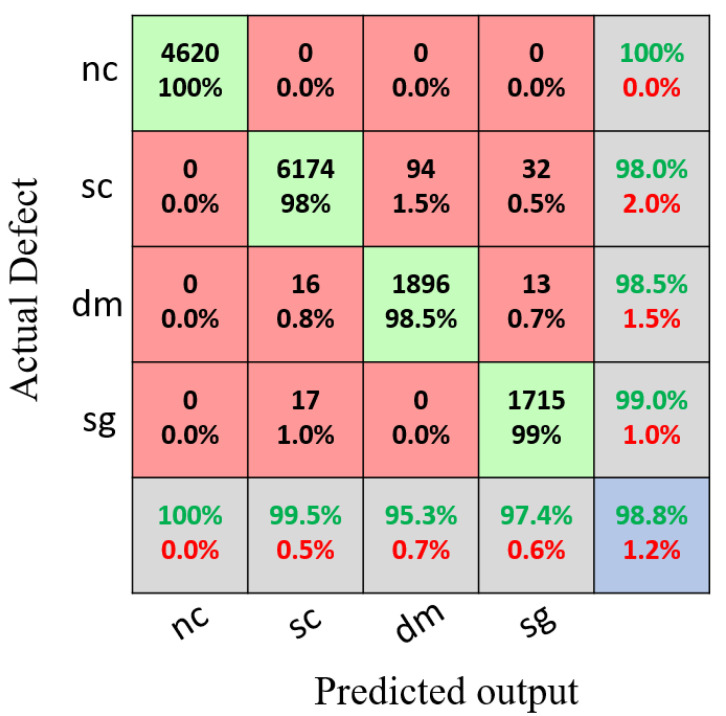
Results of the confusion matrix for the multivariant defects and non-crack concrete using the training set.

**Figure 14:**
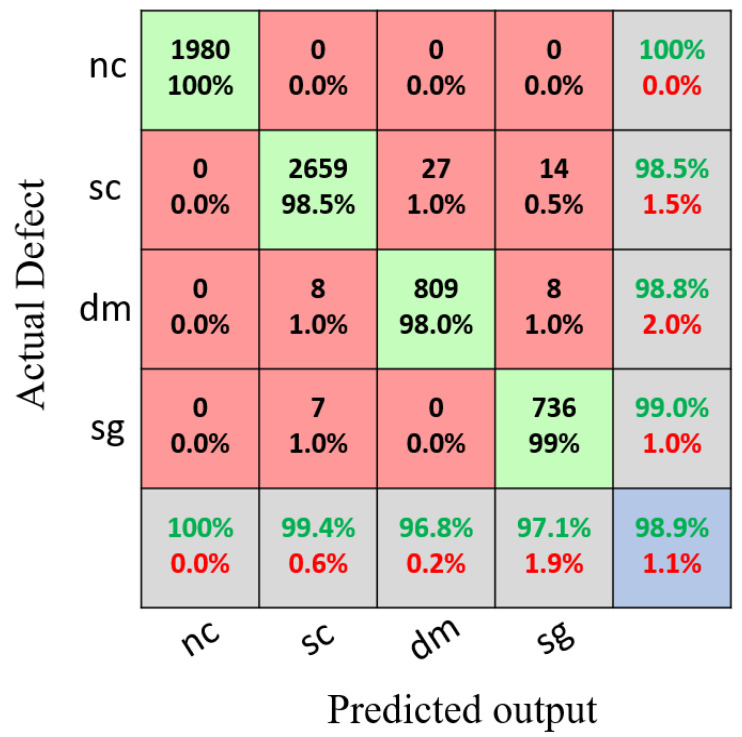
Results of the confusion matrix for the multivariant defects and non-crack concrete using the testing set.

Figure 13 shows the confusion matrix for the training set using the proposed model with a classification accuracy of 98.8%. Meanwhile, Figure 14 shows the confusion matrix for the testing set using the proposed model with a classification accuracy of 98.9%.

The experimental setup for this study compared ResNet50, VGG16, and MobiNetV2 with the proposed model. As shown in Table 4, the training and testing accuracy results of the proposed model are much higher than those of the other CNN models.

Normally, hyperparameters are particularly sensitive when training using convolutional neural networks; this study employs 5000 epochs with a learning rate of 0.0001 to assess the trained model. To ensure the experimental model performed optimally, we monitored the validation loss every epoch and modified the weight variables accordingly when the validation loss reduced during the training process.

The tables below provide the precision, recall, and F1-Score results of the experiments. The experimental findings indicate that the suggested model attained an accuracy of 98.9% for the testing dataset, which is the highest rate of damage recognition among the experimental models.

Likewise, each condition/variant of the concrete images was tested to determine the results of the different parameters used. Similarly, the proposed model was compared with the other CNN models, as shown in Table 5, Table 6, Table 7 and Table 8.

**Figure 15:**
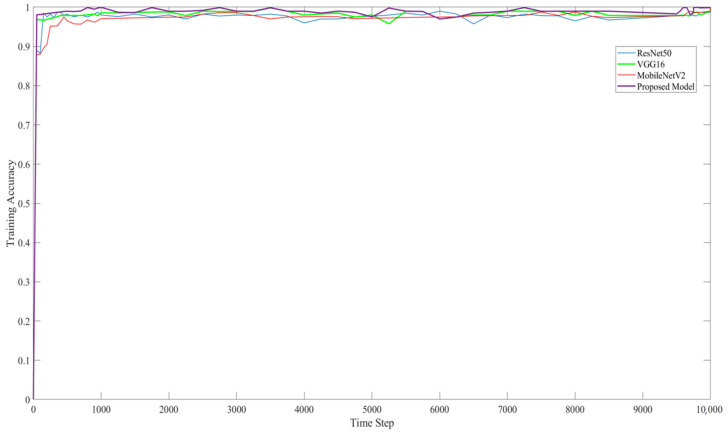
Training accuracy of ResNet50, VG16, MobileNetV2, and the proposed model on image classification.

**Figure 16:**
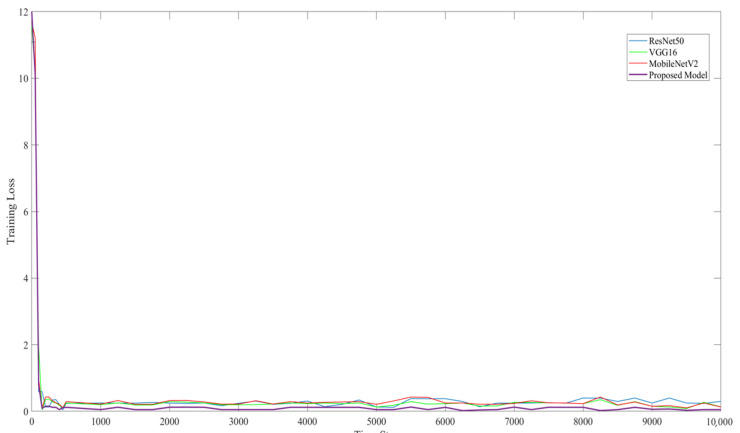
Training loss of ResNet50, VG16, MobileNetV2, and the proposed model on image classification.

Figure 15 and Figure 16 show the training accuracy and training loss, respectively, of the proposed model. They show that the proposed model provided better detection accuracy results and minimal loss.

The following is a comparative analysis of this study to the other existing relative approaches. Reference [44] uses the concept for crack detection, which is accomplished by the use of a deep fully convolutional network. The VGG16 neural network was chosen as the backbone of the FCN encoder for crack image categorization. The network makes use of an encoder to analyze an input image and extract the features required for semantic segmentation. The model that was utilized has an average accuracy of around 90%. As a remark in a normal scenario, if an image contains crack-like features/characteristics, image enhancement or filtering is required to minimize some of the extraneous images. The application is confine to one type of defect, the surface crack. The research can enhance the approach for use with other concrete defects.

As presented in [45], the application uses a deep learning framework addressing the efficient training and deployment of an automatic defect detection system and uses ResNet as the classifier, achieving an accuracy of detection at 87.5%; however, the datasets used are just limited to a total of 603 raw images. Moreover, the description of the selected concrete defects as well as the breakdown of each kind are not been discussed in any details in this paper; the efficiency of performance can be increased if the dataset is augmented.

The study in reference [46] utilizes deep neural networks to detect surface defects of concrete bridges. Additionally, the acquired dataset used a light detection and ranging scanner. Although this scheme achieved an accuracy of 90% rate, the acquisition of the presented dataset was not clearly discussed and the concept can be improved by providing details of the technical specification of the set-up.

The previous study on [47] uses machine learning to assist in determining the presence and location of cracks in concrete using surface images. The method provides a crack candidate region to categorize cracks and non-cracks. However, the accuracy detection method was not specified in order to validate the suggested model, and the dataset specifications were not supplied in a clear manner. Additionally, the annotation was not explained in detail.

The given study in [13] that employs a wavelet-based multiresolution analysis of ultrasonic signals in conjunction with the automated identification through artificial neural networks (ANNs) based on CNN has a result of an accuracy around 98%. However, the presented approach was used only on surface cracks; therefore, the performance cannot be sustained when applied to the other structural defects.

From the study in [48], the approach that employs a Mask R-CNN to localize cracks on concrete surfaces obtained an accuracy of around 93.94% in the detection of cracks on concrete surfaces; however, in order to determine other concrete defects types, the suggested network can be retrained on a broader and more diversified dataset that includes additional variants of defects.

The approach in [49] examines a variety of pre-trained CNN models for crack identification purposes, including MobileNetV2, ResNet101, VGG16, and InceptionV2 CNN models, but focuses on the MobileNet model, which achieves a 99.59% performance; however, despite the fact that the presented study includes images of walls, sidewalks, and a bridge, the concentration of the application is focused solely on surface cracks.

Lastly, the proposed scheme focuses on multivariant concrete defects such as surface cracks, delamination, and spalling. The proposed CNN model uses an integrated pooling module to minimize the loss of some of the significant information in the dataset, while the attention-based method improves some of the features of input data, but not all of it. The network pays more attention to the small but significant region of the data. The accuracy of defect detection achieves a rating of 98.8%.

## 5. Conclusions

Human intervention limits the accuracy of image evaluation. Typically, the resulting image quality evaluation is subjective rather than objective. Subjective image quality evaluations are a technique that is based on how humans perceive and evaluate image quality. Structure evaluation is vital, as human perception is not always dependable. The purpose of this study is to develop an objective structural monitoring system that will help prevent future occurrences that might cause damage to the structure as well as human injury. To address this drawback, the proposed scheme of this study aims to develop an automated image-based concrete condition identification technique capable of categorizing non-defective concrete into defective concrete and recognizing multivariant defects such as surface cracks, delamination, and spalling.

The proposed multivariant defect detection neural network architecture is based on a convolutional model capable of detecting various types of defects in concrete. Additionally, the system aims to aid in the development of different fault detection and identification techniques and speed up the assessment of the conditions of existing structural components. Experiments with various images show that the method presented is effective. The proposed model showed a classification accuracy of 98.8% for the training set and 98.9% for the testing set. Overall, it provided results on different metrics of performance.

In the future, we intend to incorporate the concept of spatial resolution into our method, which will enable us to detect microcracks in low-light conditions or to deal with a variety of external factors such as varying lighting conditions and variations in the concrete surface. Furthermore, other forms of damaged concrete, such as rebars and blistering, may be incorporated into this model in order to broaden the scope of the characteristics of the suggested neural network/model.

## Figures and Tables

**Table 1:** Breakdown of the dataset used in this study.

	Non-Crack	Surface Crack	Delamination	Spalling	Total Number of Images
Original acquired image	1200	1500	500	450	3650

**Table 2:** Comparison of the factors of machine and deep learning.

Factors	Deep Learning	Machine Learning
Data requirements	Ample amount of data is necessary	Training on fewer data is possible
Accuracy	Ensures high accuracy	With lower accuracy
Training time	Consume longer time to train	Requires less time to train
Dependence on hardware	Sometimes, a GPU is necessary for training	Training using a CPU is feasible
Hyperparameter configuration	Can be reconfigured in various ways	With limited reconfiguration capabilities

**Table 3:** Hardware specifications of the neural network computer.

Item	Specification Details
OS	Microsoft Windows 10 Pro, 64-bit
CPU	Intel(R) Core (TM) i7-1065G7
RAM	32.0 GB
GPU	NVidia GTX1080-Ti 11G

**Table 4:** Training and testing accuracy results of the other CNN models and the proposed model.

	Training Set	Testing Set
Model	Accuracy	Accuracy
ResNet50	95.7%	95.4%
VGG16	95.0%	97.3%
MobileNetV2	97.6%	97.5%
Proposed model	98.8%	98.9%

**Table 5:** Overall precision, sensitivity, and F1-Score of the non-crack concrete.

Non-Crack (nc)	Training Set			Testing Set		
Model	Precision	Sensitivity	F1-Score	Precision	Sensitivity	F1-Score
ResNet50	97.7%	98.7%	98.2%	99.4%	98.3%	98.8%
VGG16	99.0%	99.9%	99.4%	98.4%	100%	99.2%
MobileNetV2	98.6%	92.9%	95.7%	97.3%	98.5%	97.9%
Proposed model	100%	100%	100%	100%	100%	100%

**Table 6:** Overall precision, sensitivity, and F1-Score of the surface crack concrete.

Surface Crack (sc)	Training Set			Testing Set		
Model	Precision	Sensitivity	F1-Score	Precision	Sensitivity	F1-Score
ResNet50	99.3%	97.6%	98.4%	98.3%	97.3%	97.8%
VGG16	98.6%	98.9%	98.7%	99.4%	99.1%	99.2%
MobileNetV2	98.5%	98.3%	98.4%	100%	99.1%	99.5%
Proposed model	99.5%	98.0%	98.7%	99.4%	98.5%	98.9%

**Table 7:** Overall precision, sensitivity, and F1-Score of the delamination concrete.

Delamination (dm)	Training Set			Testing Set		
Model	Precision	Sensitivity	F1-Score	Precision	Sensitivity	F1-Score
ResNet50	96.7%	94.7%	95.7%	95.3%	94.3%	94.8%
VGG16	97.0%	95.6%	96.3%	97.5%	96.0%	96.7%
MobileNetV2	93.6%	98.3%	95.9%	96.3%	98.5%	97.4%
Proposed model	95.3%	98.5%	96.9%	96.8%	98.8%	97.8%

**Table 8:** Overall precision, sensitivity, and F1-Score of the spalling concrete.

Spalling (sg)	Training Set			Testing Set		
Model	Precision	Sensitivity	F1-Score	Precision	Sensitivity	F1-Score
ResNet50	94.3%	95.3%	94.8%	96.8%	98.8%	97.8%
VGG16	95.6%	98.6%	97.1%	96.3%	97.6%	96.9%
MobileNetV2	96.8%	95.8%	96.3%	96.4%	98.8%	97.6%
Proposed model	97.4%	99.0%	98.2%	97.1%	99.0%	98.0%

## Data Availability

Data available on request due to restrictions. The data presented in this study are available on request from the corresponding authors. The data are not publicly available due to the project’s contract.
